# Relationship between Lean Tissue Mass and Muscle Function in Women with Obesity

**DOI:** 10.3390/nu15214517

**Published:** 2023-10-25

**Authors:** Laurent Maïmoun, Elise Bourgeois, Chris Serrand, Thibault Mura, Jean-Paul Cristol, Justine Myzia, Antoine Avignon, Denis Mariano-Goulart, Ariane Sultan

**Affiliations:** 1 Depatment of Nuclear Medicine, CHU Montpellier, 34295 Montpellier, France; d-mariano_goulart@chu-montpellier.fr; 2 Physiology and Experimental Medicine of the Heart and Muscles (PhyMedExp), University of Montpellier, 34295 Montpellier, France; elise.bourgeois@chu-montpellier.fr (E.B.); jp-cristol@chu-montpellier.fr (J.-P.C.); a-sultan@chu-montpellier.fr (A.S.); 3 Department of Endocrinology, Nutrition and Diabetes, Team Nutrition, Diabetes, CHU Montpellier, 34295 Montpellier, France; a-avignon@chu-montpellier.fr; 4 Department of Biostatistics, Epidemiology, Public Health and Innovation in Methodology, CHU Nîmes, 30029 Nîmes, France; chris.serrand@chu-nimes.fr (C.S.); thibault.mura@chu-nimes.fr (T.M.); 5 Departement of Biochimy, Chu Montpellier, 34295 Montpellier, France; 6 Department of Clinical Physiology, CHU Montpellier, 34295 Montpellier, France; j-myzia@chu-montpellier.fr; 7 Desbrest Institute of Epidemiology and Public Health, IDESP UMR UA11 INSERM, University of Montpellier, 34295 Montpellier, France

**Keywords:** sarcopenic obesity, lean tissue mass, physical performance, muscle strength, muscle function, women with obesity

## Abstract

It is well documented that lean tissue mass (LTM) decreases with aging in patients with obesity, but there is no information available regarding muscle strength changes, a parameter that may be better associated with sarcopenic obesity (SO). The objectives of this study were to analyze the changes in LTM and fat mass (FM), muscle strength and muscle function with aging in women with obesity and to determine the prevalence of SO. LTM and FM were determined by DXA, muscle strength with the hand-grip test and muscle function with the 6 min walk test (6MWT) in 383 women with obesity. A redistribution of the LTM and FM occurred with age, characterized by a gain at the trunk to the detriment of the lower limbs, thus reducting in appendicular LTM indices. The physical performances evaluated by the muscle strength and muscle function decreased concomitantly, and the prevalence of low values for both these parameters was 22.8% and 13.4%, respectively, in the older patients. In summary, although a reduction in appendicular LTM and muscle performances occurred with age and resulted in an increase in the prevalence of SO, the number of women with obesity affected by SO remained low (*n* ≤ 15), even in those older than 60 years.

## 1. Introduction

It has been well documented that a gradual increase in body fat associated with losses in muscle mass and strength occurs with aging [1,2,3,4]. This body composition change increases the risk of both obesity and sarcopenia, which can occur simultaneously and synergistically aggravate each other and is defined as sarcopenic obesity (SO) [5]. Moreover, patients with SO were shown to be 2.5 times more at risk of disability than patients with sarcopenia or obesity alone [6]. SO has several negative consequences for health—including poor quality of life, physical disability, increased risk of fall, cardiovascular diseases, and institutionalization—resulting at term in an increased risk of early mortality [7]. In addition, low muscle mass is masked by obesity, making its diagnosis a challenge [8]. To improve the detection and medical management of these patients, a recent consensus statement from the European Association for the Study of Obesity (EASO) and the European Society for Clinical Nutrition and Metabolism (ESPEN) defined SO as an increase in body mass index (BMI) or waist circumference and the simultaneous occurrence of increased fat mass (FM), low muscle mass and low muscle strength and function [9]. However, the SO prevalence varies widely (ranging from 0% to 94%) with the criteria used [10,11], which suggests the need for consensual criteria [10] and improved methods of identifying and treating affected patients [9] in routine care settings [12]. In two recent studies [13,14], we confirmed a wide range of low muscle mass values (0 to 29.2%) when the usual cut-offs currently used were applied in older women with obesity. Although a reduction in appendicular lean tissue mass (LTM) was observed in the women suffering from obesity with aging [13,14], no older women were diagnosed with low LTM using the majority of the current cut-offs. New cut-offs developed from young women with obesity with the same disease and from the same country appeared to be better adapted [14]. Indeed, using this approach, the low LTM prevalence was relatively homogenous (8.5–17.4%). Unfortunately, in this previous study, we evaluated sarcopenia through muscle mass only, and no information was available on muscle strength, a parameter that may be better associated with muscle functional decline [14]. Consequently, before using these new cut-offs in clinical routine, their effectiveness must be evaluated, as well as whether they are associated with physical disabilities and muscle strength, to better identify subjects with obesity with SO.

The three aims of this study were: (i) to compare LTM and fat mass (FM) in women with obesity in different age groups and determine the prevalence of low LTM by using the cut-offs commonly applied in the general population and those more especially adapted to patients with obesity, (ii) to compare the muscle strength and muscle function in these age groups, and (iii) to determine the factors influencing physical performances.

## 2. Materials and Methods

### 2.1. Subject

Patients with obesity (body mass index (BMI) ≥ 30 kg/m^2^) [15] were recruited for medical care for their obesity. Only women were recruited in this study because they constituted the majority of patients seen in our department. Study patients were subdivided into three groups according to age: young patients (≤35 years), middle-aged patients (>35 to 60 years), and older patients (>60 years). The choice of the three age-group categories was based on our previous study, which allowed us to determine new cut-offs for low LTM specifically adapted to women with obesity [14].

As previously described [13,14], the exclusion criteria were the absence of bariatric surgery and any physical handicap (amputation, prosthesis, difficulties in walking) that might impede body composition measurement and muscle performance evaluations. No patient was pregnant. The medical history (menopausal status, smoking status, diabetes mellitus and medications) was also recorded. Only leisure physical activities (<1 h/week) were performed by the patients.

Standing height and weight were measured to calculate BMI [weight/height^2^ (kg/m^2^)]. Waist circumference was measured to the nearest 0.1 cm midway between the last rib and the crest of the ileum.

Type 2 diabetes [16] and arterial hypertension (HTA) [17] were defined as comorbidities.

#### Participant Consent

The study was approved by the local ethics committee (NDC-2009-1052) and performed in accordance with the ethical standards laid down in the 1964 Declaration of Helsinki and its later amendments. All participants gave written informed consent.

### 2.2. Methods

#### 2.2.1. Determination of LTM and FM

Dual-energy X-ray absorptiometry (DXA) (Hologic Horizon A, Hologic, Inc., Waltham, MA, USA) was used to determine FM (kg and %) and LTM (kg) following the procedure previously described in detail [18]. The regional soft tissue composition (upper limbs, lower limbs and trunk) was obtained from the whole-body scan. Quality control was checked every day by analyzing a lumbar spine phantom. The coefficient of variation (CV) was <1% for FM and LTM.

#### 2.2.2. Cut-Offs Used for the Definition of Low LTM

Appendicular lean mass (ALM; kg) was defined as the sum of the LTM of the upper and lower limbs [19], and the ALM/height^2^ [ALMI(h^2^); kg/m^2^] or ALM/body mass index [ALMI(BMI)] defined the ALM index. Low LTM was defined as follows: first, ALM < 15 kg and ALMI(h^2^) < 5.5 kg/m^2^, as defined by the European Working Group on Sarcopenia in Older People 2 (EWGSOP2) [20]; Second, ALM < 15.02 kg and ALMI(BMI) < 0.512, as defined by the Foundation for the National Institutes of Health (FNIH) [21]; third, ALMI(h^2^) < 5.67 kg/m^2^ [22], as defined by the International Working Group on Sarcopenia (IWGS); fourth, recently and specifically developed cut-offs for patients with obesity, which included ALM < 18.51 kg, ALMI(h^2^) < 7.15 kg/m^2^, ALMI(BMI) < 0.483; and last an obesity index calculated as T-score = [ALMI(h^2^) − (2.08 + 0.183 × BMI)]/0.72 [14]. All these cut-offs are adapted to Caucasian women.

#### 2.2.3. Assessment of Muscle Performance

Muscle strength was determined with the hand-grip test using a hand dynamometer (EH101; Zhongshan Camry, Zhongshan, China). Hand-grip strength (HGS) was measured with the participant in a standing position with the arms close to the body and the elbow at 90° flexion. Three measurements were performed for the dominant hand. The mean value was calculated and was used for analysis. One minute between each repetition was respected to avoid fatigue. Quality control of the dynamometer was ensured by routinely checking with the known values of the resistors. In women, a value <16 kg was considered low for muscle strength [23], in line with the recommendations of the EWGSOP2 [20].

Muscle function was determined with the 6 min walk test (6MWT) to evaluate aerobic endurance according to the recommendations [24]. The patients were asked to walk for 6 min as fast as possible on a 30 m shuttle track. The distance (m) covered in 6 min was recorded. The gait speed (m/s) was calculated as the distance (m) covered in 6 min. A gait speed <0.8 m/s was defined as a low value [25].

#### 2.2.4. Sarcopenic Obesity Definition

Patients were diagnosed with sarcopenic obesity if they had (i) BMI > 30 kg/m^2^; (ii) low LTM determined by DXA according to one of the following criteria (ALM < 15 kg, ALM< 15.02 kg, ALM < 18.51 kg, ALMI(h^2^) < 5.5 kg/m^2^, ALMI(h^2^) < 5.67 kg/m^2^, ALMI(h^2^) < 7.15 kg/m^2^, ALMI(BMI) < 0.512, ALMI(BMI) < 0.483, or an obesity index calculated as T-score = [ALMI(h^2^) − (2.08 + 0.183 × BMI)]/0.72 [14,20,21,22]; and (iii) either low muscle strength (<16 kg) determined by dynamometer [20] or low muscle function (<0.8 m/s) determined by 6MWT [25], as recommended by EWGSOP2 [20].

### 2.3. Statistical Analysis

Patient characteristics are described as proportions for categorical variables and as means ± standard deviations (SD) for quantitative variables. Comparisons between age groups for quantitative variables were made using either ANOVA or the Kruskal–Wallis depending on the identified distribution. Two-by-two group comparisons were also made using either the Student’s *t*-test or the Mann–Whitney U-test. For qualitative variables, the Chi2 test or the Fisher exact test were used. To account for multiple comparisons and the inflation of risk alpha, we corrected the estimated *p*-value through the Bonferroni procedure. Correlations between parameters were estimated through the Spearman correlation coefficient and graphically through a scatter plot. Finally, in order to determine whether the effect of age on grip strength and gait speed was mediated by the decrease in ALMI(BMI) with age, we used the CAUSALMED procedure [26] in SAS, which uses linear regression adjustment methods [27] to estimate the percentage of the total effect of age that can be attributed to the mediation by ALMI(BMI).

Statistical analyses were performed at the conventional two-tailed α level of 0.05 using SAS Enterprise Guide software version 7.1 (SAS Institute, Cary, NC, USA) or R software version 4.1.1 (R Core Team (2021), Vienna, Austria).

## 3. Results

### 3.1. Characteristics of Patients

The clinical and biological characteristics and comorbidities of the patients are presented in Table 1. A total of 383 women were recruited. Eighty constituted the young group (mean age 26.4 ± 5.2 years), 201 the middle-aged group (mean age 48.5 ± 6.8 years), and 102 the older group (mean age 66.6 ± 5.0 years). Globally, weight, height, BMI and hip circumference were higher in the young and middle-aged groups compared to the older group, whereas waist circumference was lower. Resting energy expenditure was lower in the middle-aged and older groups compared to the young group. Comorbidities increased with age, and the older group presented a prevalence of 61.8% for HTA and 40.2% for diabetes.

### 3.2. Body Composition

The young and middle-aged groups presented systematically higher absolute values for FM and LTM (upper limb FM and whole-body FM% excepted) than the older group. To take into account the differences in height and weight between groups, an adjustment for these two parameters was performed (Table 2). Whole-body LTM and FM were relatively comparable between the three groups, although a redistribution of these two components characterized by a gain at the trunk to the detriment of the lower limbs occurred with age.

### 3.3. Sarcopenic Index

For all the parameters evaluating low LTM (ALM, ALMI(h^2^), ALMI(BMI) and obesity index), lower values were found in the >60 yrs group compared to the ≤35 yrs group, while few differences were observed between ≤35 yrs and 35–60 yrs groups. The prevalence of low LTM in the three groups was calculated with the different cut-offs [20,21,22] (Table 3). A wide range of low LTM prevalence in the older group was observed, from 0 to 2% according to EWGSOP2 [ALM, ALMI(h^2^)] and IWGS [ALMI(h^2^)] to 20.6% according to FNIH [ALMI(BMI)]. The prevalence of low LTM was very limited in the young and middle-aged patients for all indices. Interestingly, the prevalence of low LTM gradually increased with age when specific cut-offs developed for women with obesity were used [14]. Moreover, for each age group, the prevalence of low LTM appeared more consistent between cut-offs (ranging from 7.9% to 18.6%) when the cut-offs developed for subjects with obesity were used compared with the cut-offs currently used for the general population.

### 3.4. Muscle Function

The physical performances determined by the hand-grip test and the 6MWT were significantly lower in the >60 yrs group compared to the <35 yrs group, while only distance and walking speed values were different between the <35 yrs and 35–60 yrs groups. When the prevalence of low values for muscle strength (<16 kg) and gait speed (<0.83 m/s) was calculated, an increase in prevalence was observed in the >60 yrs compared to <35 yrs and 35–60 yrs groups (Table 3). The prevalence of low values in the middle-aged and older groups appeared more marked for the grip test (22.8%) than for the 6MWT (13.4%).

To determine whether patients with low or high values for muscle strength and muscle function presented specific characteristics, a sub-analysis was performed according to the two respective cut-offs (Table 4). Patients with grip test results >16 kg were younger and presented higher WB FM, trunk FM and LTM at all sites (whole body, trunk, arms and legs), ALM and ALMI(h^2^), 6MWT values (gait speed and distance covered) and REE compared to patients presenting grip test values <16 kg. Regarding muscle function, although the patients presenting values >0.83 m/s were younger than those with values <0.83 m/s, interestingly, the two groups did not differ for any body composition (FM and LTM) parameters. ALMI(BMI), gait speed and grip strength were higher in patients with the higher 6MWT values.

### 3.5. Prevalence of Sarcopenic Obesity

**Figure 1:**
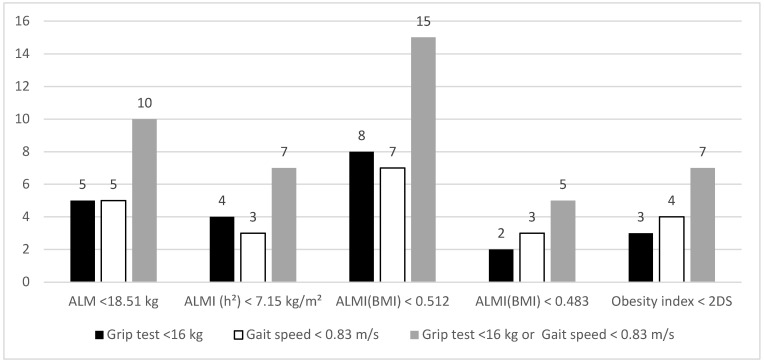
Patients presenting low muscle strength, function and mass defined by different cut-offs. Data are presented as number of patients. ALM: appendicular lean mass; ALMI: appendicular lean mass index [ALMI(h^2^): ALM/height^2^ and ALMI(BMI): ALM/BMI)]; BMI: body mass index (weight/height^2^). Obesity index was defined as T-score = [ALMI(h^2^) − 2.08 + 0.183 × BMI)]/0.72 [14].

The number of patients presenting low muscle strength and/or low physical function according to the different cut-offs for low LTM is shown in Figure 1. Whatever the cut-off used for LTM, the prevalence of SO remained low (ranging from 2 to 15 patients).

### 3.6. Impact of Age and BMI on Muscle Mass and Muscle Performance

**Figure 2:**
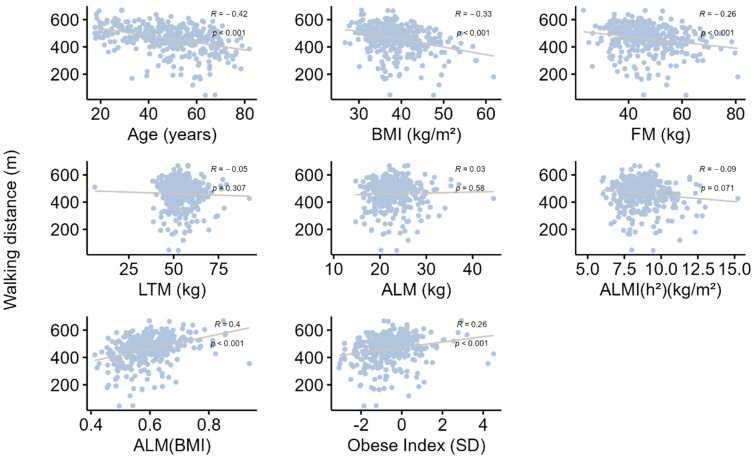
Correlation analysis between distance covered in the 6 min walking test and various parameters. Data are presented as Spearman correlation coefficients (R), and *p* indicates the degree of significance. BMI: body mass index; FM: fat mass; LTM: lean tissue mass; ALM: appendicular lean mass; ALMI: appendicular lean mass index [ALMI(h^2^): ALM/height^2^ and ALMI(BMI): ALM/BMI)]; BMI: body mass index (weight/height^2^). Obesity index was defined as T-score = [ALMI(h^2^) − 2.08 + 0.183 × BMI)]/0.72 [14]; REE: resting energy expenditure.

**Figure 3:**
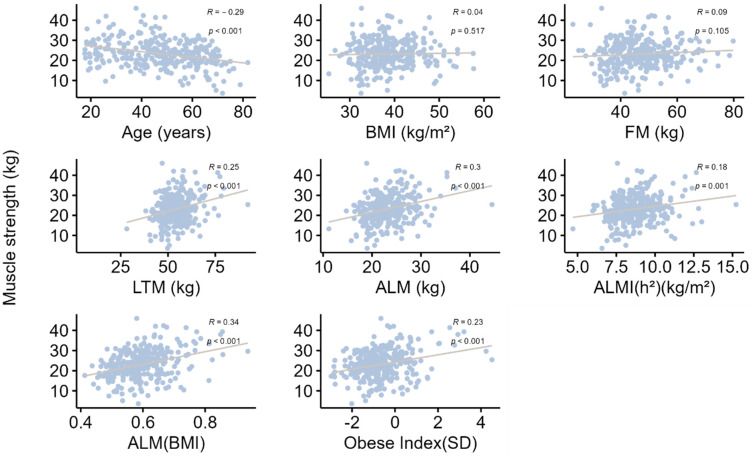
Correlation analysis between muscle strength evaluated by hand-grip test and various parameters. Data are presented as Spearman correlation coefficients (R), and *p* indicates the degree of significance. BMI: body mass index; FM: fat mass; LTM: lean tissue mass; ALM: appendicular lean mass; ALMI: appendicular lean mass index [ALMI(h^2^): ALM/height^2^ and ALMI(BMI): ALM/BMI)]; BMI: body mass index (weight/height^2^). Obesity index was defined as T-score = [ALMI(h^2^) − 2.08 + 0.183 × BMI)]/0.72 [14]; REE: resting energy expenditure.

All correlations are presented in Table 5, Figure 2 and Figure 3. Briefly, age was significantly and negatively associated with grip strength, gait speed and all muscle mass indices. The strength of these associations increased after adjustment for BMI, indicating a confounding effect of BMI, which was negatively correlated with age and gait speed and positively correlated with all muscle mass indices, but not grip strength. In addition, grip strength was positively correlated with gait speed. Moreover, grip strength and gait speed were also positively correlated with ALMI(BMI) and the Obesity Index, raising the hypothesis of a mediating role for the decrease in appendicular muscle mass relative to BMI in the decline in muscle strength with age. This hypothesis was confirmed by mediation analyses (Table 6), which found that 27.6% (SEM = 7.9; *p*-value < 0.001) of the decrease in grip strength with age and 20.7% (SEM = 5.2; *p*-value < 0.001) of the decrease in gait speed with age could be explained by the concurrent decrease in ALMI(BMI).

## 4. Discussion

In this cross-sectional study carried out on a large number of women with obesity, we found a whole-body composition redistribution of LTM and FM components with aging, leading to the lowest appendicular LTM index in the older patients. A progressive decrease in muscle strength and performance was concomitantly observed, inducing an increase in the prevalence of SO that nevertheless remained relatively low in the women with obesity, even in those over 60 years.

### 4.1. Body Composition Change with Age

In the present study, we performed an age subgroup analysis and observed a progressively increasing propensity toward central/abdominal adiposity and LTM to the detriment of appendicular sites, with the lower limbs most affected. These findings confirmed previous results in women with obesity [13,14]. The consequence of the soft tissue rearrangement, particularly for LTM, was a progressive decrease with aging in all indices used to determine low muscle mass—a parameter included in the definition of sarcopenia. Interestingly, this finding was observed whatever the index or factor of adjustment (i.e., none, h^2^ or BMI), which may have influenced the result due to the specific anthropometric characteristics of our patients. Nevertheless, when the validated cut-offs for Caucasian women were applied [20,21,22], the prevalence of low LTM presented a wide range that is highly dependent on the set of diagnostic criteria, confirming previous findings [10,11,13,14]. However, when the results were examined in greater detail, only a minority of patients (0% to 2%) was identified as having low LTM when ALM and ALMI(h^2^) were applied, whereas ALMI(BMI) seemed to overestimate the prevalence at 20.6%. In subjects with obesity aged from 60 to 99 years, a prevalence of low LTM ranging from 0.2% to 4% according to the cut-off used was reported [28]. Given the inconsistency of the results with the various cut-offs [10,11,29]—probably due to their unsuitability for this population with obesity—we used secondary new cut-offs recently developed from data obtained in a population of young French women with obesity [14]. The results obtained with this method revealed a higher low LTM prevalence in women with obesity older than 60 years. Moreover, the prevalence of low LTM appeared more consistent across the different cut-offs (ranging from 7.9% to 18.6%).

### 4.2. Variation in Muscle Function with Age

It is now well acknowledged that muscle mass alone is insufficient to diagnose sarcopenia. It should first be determined by a deterioration of muscle strength (dynapenia), and its level of severity should be evaluated by physical performance testing [20]. Nevertheless, we can note that the relationship between strength and mass generally appeared to be non-linear [20]. One question arose from our observations: Is the increase in low LTM prevalence after 60 years associated with a deterioration in muscle function with aging in patients with obesity? We found in our population a concomitant and gradual reduction in muscle strength with aging determined by the hand-grip test and muscle performance determined by the 6MWT, the two tests recommended by the EWGSOP2 guidelines for determining sarcopenia [20]. Moreover, these tests are highly reproducible in subjects with obesity and can be used in clinical routine [30]. To the best of our knowledge, only Otten et al. [29] similarly reported a reduction in hand grip and knee extension strength with age in women with obesity (mean BMI 43.5 kg/m^2^, age ranging from 18–78 years). In this previous study, LTM was also positively correlated with the strength parameters, whereas BMI and FM were not. Similarly, in a population of subjects with obesity ranging from 19 to 80 years, 6MWT appeared negatively correlated with age [31].

We also observed that 22.8% of the patients older than 60 years presented altered muscle strength and only 13.4% altered muscle performance. The preponderance of muscle strength alteration was somewhat unexpected since a high BMI in subjects with obesity should have the greatest impact on walking capacity compared to the general population. Correlation analysis confirmed our hypothesis that BMI would be inversely correlated with gait speed, whereas no correlation was found for hand-grip strength. Previous studies have also reported that 6MWT results were influenced by various factors such as disabilities, but mainly by the severity of obesity [30,31]. Similarly, Purcell et al. [11] recently reported that, although SO increased across age categories in a cohort of older Canadian adults (age > 65 years, *n* = 11,803, 50.4% women), it was mainly associated with low hand-grip strength, but not with slow gait speed. Kong et al. [32] also observed in elderly South Korean subjects that the group with SO tended to have lower grip test values than the normal, pure obesity, and pure sarcopenia groups, but gait speed was not different between groups. Conversely, a positive association between BMI and hand-grip strength was also reported in a group composed of normal and overweight adults [33]. In this previous study, Keevil et al. [34] noted no increase in grip strength beyond a BMI ≥ 30 kg/m^2^, which may explain why no correlation between BMI and strength was observed in our obese population [29].

In fact, our results tended to show that some of the data on patients’ muscular capacities provided by the hand-grip and 6MWT tests are common, as shown by (i) the positive correlation observed between the two tests (r = 0.360, *p* < 0.001) and the previously reported [31], and (ii) the comparable age of the participants presenting values that were lower and higher (respectively, 57–58 years and 46.6–48) than the cut-off points for the two tests (i.e., 16 kg and 0.83 m/s). Nevertheless, it was also interesting to note some discordance between the two tests: (i) different factors were associated with each of them: the grip test was positively linked to LTM (whole body and regional), whereas the walking test was only negatively correlated with FM (whole body and regional), and (ii) when subgroup analysis was performed according to the cut-offs, patients with values >16 kg presented significantly higher LTM values than those with values <16 kg, whereas LTM values were comparable between patients with values higher and lower than 0.83 m/s, suggesting more an alteration of muscle quality. Barrea et al. reported that subjects with obesity with ages ranging from 18 to 51 years and grip test values <16 kg also exhibited a lower LTM value compared with their counterparts, with values above the cut-off [35]. However, in this study, body composition was analyzed with bioelectrical impedance analysis (BIA), a technique less accurate technique than DXA [33].

Our findings suggested a preponderant relationship between muscle mass and muscle strength, although high adiposity or muscle quality deterioration has appeared as the main predictor of muscle performance limitations in older adults [11,36]. Interestingly, our mediation approach showed that the decrease in muscle strength observed with aging was mediated by a nearly 27.6% decrease in LTM. Nevertheless, the proportion of the effect on muscle strength mediated by muscle mass remained moderate, suggesting that other factors may affect strength parameters in subjects with obesity. Also, impaired muscle quality partly due to fat infiltration of skeletal muscles, known as myosteatosis, causing changes in muscle tissue composition and metabolic efficiency or low-grade inflammation, has been proposed as a contributing deleterious factor [29,37,38]. Due to these tissue alterations, the deterioration in physical function may be greater in patients with SO than in those with pure sarcopenia [32].

Finally, our results indicated that the prevalence of SO was relatively low in our population, less than 9% for most of the cut-offs used to define low LTM. It is currently estimated that from 2.6% to over 90% of older adults globally present SO using various definitions, but generally the prevalence remains low until increasing dramatically after the age of 70 years [10,25,39,40,41]. The limited mean age of our older patients (66.6 years) may explain the relatively lower SO observed in our study. Moreover, as expected, although our older group was the most affected by SO, younger patients may also develop it due to sedentary lifestyles and unhealthy diets [42,43]. It is also likely that the cut-offs used to define low muscle function are not adapted to the population with obesity. El Gogh et al. [44] found that the cut-offs to discriminate patients with normal and low LTM were 23.5 kg for the hand-grip test and 1.2 m/s for gait speed. However, we note that these values were determined from a population of women with obesity that presented an unexpectedly high prevalence (63.3%) of low LTM, and consequently these cut-offs were not appropriate for our population.

### 4.3. Strengths and Limitations of the Study

This study presents numerous strengths. To our knowledge, this is the first cross-sectional analysis of the variation in prevalence with age of SO among a population with obesity in France using the EWGSOP2 definition of sarcopenia and a specifically adapted definition for women with obesity. Moreover, the DXA technique, which is considered the gold standard technique for clinical body composition evaluation in subjects with obesity was used [45]. All the patients were Caucasian, thus avoiding the potential effects of ethnicity on body composition and sarcopenia prevalence. Last, all the patients were recruited from one center, which limited the differences among investigators about the way in which physical function and body composition are measured. The cross-sectional design may be the main limitation of our study as it did not allow us to follow the concomitant variations in muscle mass and muscle performance with age in the same subjects, thus introducing the likelihood of inter-individual variability. However, the wide age range of these patients with obesity may offer a practical method to assess the prevalence variation with age in the general population and, by extension, in subjects with obesity. In the future, our results should nevertheless be confirmed in a longitudinal study, which would also provide more precision on the gradual changes that occur due to aging. The prevalence of SO was low in our population, which may have limited the possibility of identifying other factors associated with this disease in this population.

## 5. Conclusions

In conclusion, our results suggest that, with aging, women with obesity present an increase in truncal LTM and FM to the detriment of the lower limbs, leading to a lower ALM index at an older age. A progressive decrease in muscle performance (strength and function) was concomitantly observed. The conjunction of muscle mass and muscle performance deterioration resulted in an increase in the prevalence of SO, which nevertheless remained low in these subjects with obesity, even in those older than 60 years. Muscle mass rather than BMI or FM was positively correlated with muscle strength. The evidence of a moderately mediated effect of muscle mass should encourage us to look for other clinically measurable parameters associated with muscle strength.

## Figures and Tables

**Table 1:** Characteristics of women with obesity regarding age group.

	Women with Obesity (Class of Age)			
	≤35 Yrs (*n* = 80)	35–60 Yrs (*n* = 201)	>60 Yrs (*n* = 102)	*p*-Value
Age, years	26.4 ± 5.2 ^a^	48.5 ± 6.8 ^b^	66.6 ± 5.0 ^c^	**<0.01**
**Clinical characteristics**				
Weight, kg	104.8 ± 16.6 ^a^	104.0± 15.8 ^a^	96.2 ± 13.5 ^b^	**<0.01**
Height, m	163.4 ± 6.3 ^a^	162.7 ± 6.3 ^a^	160.1 ± 5.6 ^b^	**<0.01**
BMI, kg/m^2^	39.2 ± 5.8 ^a^	39.4 ± 6.0 ^b^	37.5 ± 4.5 ^a^	**0.02**
Waist circumference, cm	105.4 ± 12.3 ^a^	109.8 ± 12.2 ^b^	110.1 ± 11.6 ^b^	**0.04**
Hip circumference, cm	124.8 ± 11.9 ^b,c^	125.9 ± 12.8 ^a,c^	121.1 ± 11.5 ^b^	**0.02**
**Comorbidities**				
HTA, *n*; %	4 (5) ^a^	72 (35.8) ^b^	63 (61.8) ^c^	**<0.01**
Diabetes, *n*; %	5 (6.3) ^a^	45 (22.4) ^b^	41 (40.2) ^c^	**<0.01**
**Metabolism parameter**				
REE, cal/24 h	1802.3 ± 249.9 ^a^	1739.1 ± 288.3 ^a^	1622.5 ± 243.9 ^b^	**<0.01**

Data are presented as the mean ± standard deviation. BMI: body mass index (weight/height^2^); HTA: arterial hypertension; *n*: number, %, percentage; REE: resting energy expenditure. Groups presenting the same letter (a, b or c) are not different.

**Table 2:** Lean tissue mass and fat mass in women with obesity regarding the age group adjusted on height and weight.

	Women with Obesity (Class of Age)			
	≤35 Yrs (*n* = 80)	35–60 Yrs (*n* = 201)	>60 Yrs (*n* = 102)	*p*-Value
**Body composition**				
**Fat mass**				
Whole body, kg	47.2 ± 6.9	46.8 ± 4.3	47.0 ± 6.2	0.725
Whole body, %	45.4 ± 5.6	45.1 ± 3.5	45.1 ± 5.1	0.800
Trunk, kg	21.9 ± 4.9 ^a^	23.1 ± 3.1 ^b^	23.9 ± 4.4 ^c^	**<0.001**
Upper limbs, kg	5.8 ± 3.3	5.9 ± 2.1	6.4 ± 3.0	0.090
Lower limbs, kg	18.5 ± 5.2 ^a^	16.9 ± 3.3 ^b^	15.8 ± 4.7 ^c^	**<0.001**
**Lean tissue mass**				
Whole body, kg	53.8 ± 7.2	53.9 ± 4.5	54.4 ± 6.5	0.630
Trunk, kg	27.0 ± 4.9 ^a^	28.1 ± 3.1 ^b^	28.9 ± 4.4 ^c^	**0.0002**
Upper limbs, kg	5.2 ± 1.2	5.3 ± 0.8	5.2 ± 1.2	0.720
Lower limbs, kg	18.4 ± 2.6 ^a^	17.6 ± 1.6 ^b^	17.3 ± 2.4 ^b^	**<0.0001**

Data are presented as the mean ± standard deviation. Groups presenting the same letter (a, b or c) are not different.

**Table 3:** Sarcopenic index in women with obesity regarding age group.

	Women with Obesity (Age Class)			
	≤35 Yrs (*n* = 80)	35–60 Yrs (*n* = 201)	>60 Yrs (*n* = 102)	*p*-Value
**Low lean tissue mass**				
ALM. kg	24.24 ± 3.43 ^a^	23.27 ± 4.01 ^a^	21.24 ± 3.20 ^b^	**<0.01**
ALM < 15 kg (*n*, %)	0 (0)	1 (0.5)	2 (2.0)	0.49
ALM < 18.51 kg (*n*, %)	2 (2.5) ^a^	19 (9.5) ^a,b^	19 (18.6) ^b^	**<0.01**
ALMI(h^2^), kg/m^2^	9.07 ± 1.15 ^a^	8.78 ± 1.40 ^a^	8.27 ± 1.03 ^b^	**<0.01**
ALMI(h^2^) < 5.5 kg/m^2^ (*n*.%)	0 (0)	1 (0.5)	0 (0)	1
ALMI(h^2^) < 5.67 kg/m^2^ (*n*.%)	0 (0)	1 (0.5)	0 (0)	1
ALMI (h^2^) < 7.15 kg/m^2^	0 (0) ^a^	16 (8) ^b^	13 (12.8) ^b^	**<0.01**
ALMI(BMI)	0.622 ± 0.083 ^a^	0.603 ± 0.076 ^b^	0.570 ± 0.074 ^c^	**<0.01**
ALMI(BMI) < 0.512	5 (6.3) ^a^	25 (12.4) ^a,b^	21 (20.6) ^b^	**0.02**
ALMI(BMI) < 0.483	3 (3.8)	8 (4.0)	8(7.9)	**0.32**
Obesity index *	−0.26 ± 1.06 ^a^	−0.68 ± 1.05 ^b^	−0.93 ± 1.00 ^b^	**<0.01**
Obesity index * (<2DS)	3 (3.8)	17 (8.5)	13 (12.8)	**0.1**
**Physical performance**				
6-min walking test, m	511.9 ± 76.9 ^a^	464.5 ± 93.1 ^b^	410.4 ± 105.0 ^c^	**<0.01**
Walking speed m/s	1.42 ± 0.21 ^a^	1.29 ± 0.26 ^b^	1.14 ± 0.29 ^c^	**<0.01**
Walking speed < 0.83 m/s (*n*, %)	1.0 (1.3) ^a^	11 (5.6) ^a,b^	13 (13.4) ^b^	**<0.01**
Grip test, kg	24.9 ± 6.8 ^a^	23.7 ± 6.7 ^a^	20.3 ± 6.3 ^b^	**<0.01**
Grip test < 16 kg (*n*, %)	6 (8) ^a^	15 (9.4) ^a^	18 (22.8) ^b^	**<0.01**

Data are presented as the mean ± standard deviation. ALM: appendicular lean mass; *n*: number, %, percentage; ALMI: appendicular lean mass index [ALMI(h^2^): ALM/height^2^ and ALMI(BMI): ALM/BMI)]; BMI: body mass index (weight/height^2^). * Obesity index was defined as T-score = [ALMI(h^2^) − (2.08 + 0.183 × BMI)]/0.72 [14]. Groups presenting the same letter (a, b or c) are not different.

**Table 4:** Characteristics of patients according to muscle strength and muscle function cut-offs.

	Grip Test		Gait Speed	
Parameters	<16 kg (*n* = 39)	≥16 kg (*n* = 275)	<0.83 m/s	≥0.83 m/s
Age, years	57.2 ± 14.8	46.6 ± 14.8 **	58.1 ± 11.4	48.0 ± 15.0 **
BMI, kg/m^2^	37.1 ± 6.3	39.0 ± 5.4 *	41.0 ± 7.1	39.0 ± 5.6
WB FM, kg	43.2 ± 9.7	47.5 ± 9.7 **	47.58 ± 10.9	47.1 ± 9.8
Trunk FM, kg	43.3 ± 4.1	44.2 ± 4.9 *	23.7 ± 5.8	23.1 ± 5.4
Arms FM, kg	5.6 ± 1.8	6.0 ± 1.5	6.2 ± 1.7	6.0 ± 2.4
Legs FM, kg	16.3 ± 5.1	17.2 ± 4.7	16.7 ± 5.1	17.0 ± 4.8
WB LTM, kg	51.2 ± 7.2	54.9 ± 7.4 **	54.2 ± 8.4	54.2 ± 7.6
Trunk LTM, kg	26.6 ± 3.5	28.0 ± 3.9 *	29.5 ± 9.2	28.1 ± 3.8
Arms LTM, kg	5.0 ± 0.9	5.3 ± 0.9 *	5.3 ± 1.15.3 ± 3.3	5.3 ± 0.9
Legs LTM, kg	16.5 ± 3.2	18.0 ± 3.2 **	17.8 ± 3.4	17.8 ± 3.2
ALM, kg	21.5 ± 4.0	23.3 ± 3.8 **	22.6 ± 4.1	23.1 ± 3.8
ALMI(h^2^), kg/m^2^	8.37 ± 1.41	8.76 ± 1.26 *	8.9 ± 1.6	8.7 ± 1.3
ALMI(BMI),	0.580 ± 0.080	0.600 ± 0.080	0.560 ± 0.070	0.600 ± 0.080 *
6-min walking test, m	417.5 ± 108.4	474.5 ± 93.4 **	209.4 ± 67.3	478.6 ± 73.3 **
Gait speed. m/s	1.16 ± 0.30	1.32 ± 0.26 **	0.58 ± 0.19	1.33 ± 0.2 **
Grip test, kg	11.9 ± 3.05	24.7 ± 5.7 **	18.2 ± 5.3	23.5 ± 6.9 **
REE, cal/24 h	1582.3 ± 270.0	1743.8 ± 271.3 **	1745.5 ± 303.4	1724.8 ± 273.7

Data are presented as the mean ± standard deviation. * *p* < 0.05. ** *p* < 0.01. BMI: body mass index (weight/height^2^); FM: fat mass; WB: whole body; LTM: lean tissue mass; ALM: appendicular lean mass; ALMI: appendicular lean mass index [ALMI(h^2^): ALM/height^2^ and ALMI(BMI): ALM/BMI)]; BMI: body mass index (weight/height^2^). Obesity index was defined as T-score = [ALMI(h^2^) − 2.08 + 0.183 × BMI)]/0.72 [14]. Only parameters presenting differences between groups (<16 kg or ≥16 kg and <0.83 m/s or ≥0.83 m/s, respectively) are presented.

**Table 5:** Correlations between muscle strength or function with age, anthropometric values, or lean tissue mass index.

Parameters	Age	BMI	Grip Strength	Gait Speed	ALM	ALMI(h^2^)	ALMI(BMI)	Obesity Index
**Age**	-	−0.113 *	−0.292 ***	−0.417 ***	−0.354 ***	−0.283 ***	−0.317 ***	−0.284 ***
**BMI**		-	0.036	−0.330 ***	0.636 ***	0.785 ***	−0.266 ***	−0.040
**Grip strength**	−0.292 ***	0.036	-	0.369 ***	0.297 ***	0.183 **	0.339 ***	0.234 ***
**Walking distance**	−0.417 ***	−0.330 ***	0.369 ***	-	0.029	−0.094	0.398 ***	0.259 ***
**Whole body FM**	−0.170 ***	0.844 ***	0.091	−0.256 ***	0.643 ***	0.591 ***	−0.127 *	−0.181 ***
**FM arms**	−0.034	0.675 ***	0.042	−0.283 ***	0.473 ***	0.485 ***	−0.149 **	−0.117 *
**FM trunk**	−0.009	0.769 ***	0.035	−0.347 ***	0.505 ***	0.467 ***	−0.199 ***	−0.277 ***
**FM legs**	−0.334 ***	0.620 ***	0.141 *	−0.052	0.576 ***	0.508 ***	0.013	−0.029
**Whole body LTM**	−0.219 ***	0.643 ***	0.245 ***	−0.053	0.903 ***	0.754 ***	0.402 ***	0.361 ***
**LTM arms**	−0.196 ***	0.442 ***	0.244 ***	−0.032	0.738 ***	0.650 ***	0.441 ***	0.473 ***
**LTM trunk**	−0.076	0.548 ***	0.173 **	−0.096	0.693 ***	0.551 ***	0.269 ***	0.164 **
**LTM legs**	−0.372 ***	0.635 ***	0.284 ***	0.038	0.976 ***	0.839 ***	0.488 ***	0.504 ***
**REE**	−0.325 ***	0.544 ***	0.189 ***	−0.036	0.688 ***	0.579 ***	0.275 ***	0.235 ***

Data are presented as Spearman correlation coefficients. * indicates a significant correlation for *p* < 0.05. ** for *p* < 0.01. *** for *p* < 0.001. FM: fat mass; LTM: lean tissue mass; ALM: appendicular lean mass; ALMI: appendicular lean mass index [ALMI(h^2^): ALM/height^2^ and ALMI(BMI): ALM/BMI)]; BMI: body mass index (weight/height^2^). Obesity index was defined as T-score = [ALMI(h^2^) − 2.08 + 0.183 × BMI)]/0.72 [14]; REE: resting energy expenditure.

**Table 6:** Mediation effects of whole-body LTM on muscle strength and gait speed, adjusted for body mass index.

	Muscle Strength			Gait Speed		
	Effect (%)	SD	*p*-Value	Effect	SD	*p*-Value
***Proportion of the total effect of age mediated by whole-body LTM***	27.6	7.8	0.0004	20.7	5.2	<0.0001

LTM: lean tissue mass; SD: standard deviation.

## Data Availability

The data used in the present analysis can be obtained through request to the corresponding author.
